# Sex differences in ocular biometric measurements: A twin study

**DOI:** 10.3389/fmed.2022.936738

**Published:** 2022-11-17

**Authors:** Han Zhang, Jing Zhou, Lili Yang, Xiaogung Zhang, Wei Shi, Hailong Yang, Guisen Zhang, Jie She, Huixia Li

**Affiliations:** ^1^Department of Ophthalmology, Inner Mongolia Chaoju Eye Hospital, Hohhot, China; ^2^Department of Ophthalmology, Baotou Chaoju Eye Hospital, Baotou, China; ^3^Department of Ophthalmology, Hohhot Chaoju Eye Hospital, Hohhot, China

**Keywords:** adolescents, opposite-sex twin-pairs, ocular biometric measurements, refractive prediction error, same-sex twin-pairs

## Abstract

**Objective:**

Gender differences in ocular biometric measurements of opposite-sex and same-sex twin pairs are still unclear. We aimed to investigate the difference between ocular biometric measurements in adolescent twin pairs.

**Materials and methods:**

This retrospective study included a total of 64 eyes of 64 adolescents from 32 twins. The ocular biometric measurements and refractive prediction error (RE) were acquired from four groups of dizygotic (DZ) twins: boys from same-sex twin-pairs (SSM, *n* = 20), boys from opposite-sex twin-pairs (OSM, *n* = 8), girls from opposite-sex twin-pairs (OSF, *n* = 8), and girls from same-sex twin-pairs (SSF, *n* = 29).

**Results:**

The mean age of the patient was 9.92 ± 2.84 (range: 6–18) years. Overall, boys had higher height, AL, WTW, but lower Ks, and Kf than girls (*p* < 0.05). Specifically, SSF was found to have the lowest lens thickness (LT), anterior chamber depth (ACD), central corneal thickness (CCT), white to white (WTW), and axial length (AL) levels, while the highest keratometry readings in the flat (Kf) and steep (Ks) levels compared with OSM, OSF, and SSM adolescents (*p* < 0.05). Compared with the OSF adolescents, ACD levels of the SSF adolescents were significantly lower [(2.99 ± 0.35) and (3.26 ± 0.15) mm, *p* = 0.033)], but Kf indicator was significantly larger [(43.93 ± 1.64) and (42.91 ± 1.75), *p* = 0.016)].

**Conclusion:**

Our study indicates that there was a significant difference in ocular biometric measurements between twin pairs, and sharing the uterus with a DZ twin SSF has smaller ocular indicator measurements. Our findings provide information on the eyeball and refractive development in adolescents.

## Introduction

Recently, the population with myopia has been found to increaserapidly, and myopia is developing more at a younger age, establishing a great concern for the health of children and adolescents (1). There is a significant correlation between myopia and various ocular biometry parameters, such as choroidal thinning and axial elongation (2). Moreover, although the exact mechanisms are still unknown, many genes are associated with ocular biometric parameters. For example, the single-nucleotide polymorphisms (SNPs, rs1401999) in the ABCC5 gene contribute to being associated with both anterior chamber depth (ACD) (3), and rs4148568 might be associated with the axial length (AL), carriage of high-risk rs4148568 genetic variants is associated with making the AL longer in northern Chinese people (4). Additionally, ocular biometric measurements are associated with demographic, environmental, and lifestyle factors such as age, gender, and diet (5, 6).

However, there are few population-based age norms for ocular biometry, particularly for children and adolescents. Analytic methods available in the context of the classical twin design offer unique opportunities to explore this issue based on the same genetic information. In the present study, we utilized data from a retrospective study, to conduct a comprehensive, informed examination of sex differences in various ocular biometry parameters.

## Materials and methods

This is a retrospective study. In total, 32 dizygotic (DZ) twin pairs aged 6–18 years old (from grade 1 of primary school to grade 3 of senior high school) were included from January 2021 to December 2021. The mean age of twins was 9.92 ± 2.84 years old. This study included both same-sex and opposite-sex twin pairs, in order to investigate the sex difference in various ocular biometry parameters.

The sample size was calculated using STATA analysis software version 14.2. Type I error was set at alpha equal to 0.05, and the type II error was set at beta equal to 0.20. AL was set as the primary outcome, and its mean difference was 0.02 with a standard deviation (SD) of 0.03 (7). After the calculations, the minimum required number was 63 eyes.

Inclusion criteria: (1) twin pairs; (2) spherical equivalent (SE) for distance refraction less than 5 D; (3) astigmatism less than 1.5 D, and (4) a best-corrected visual acuity (BCVA) over 0.1 logarithms of the minimum angle of resolution (logMAR). Exclusion criteria: (1) with diabetes, thyroid, or any systematic diseases; (2) with glaucoma, cataract, traumas, or previous ocular surgery; or (3) with systemic disease or ocular problem that may interfere with visual acuity like corneal opacity.

Demographic information was obtained by questionnaires including age, medical status, and parental refractive status. Self-reported height was also recorded.

All individuals underwent a comprehensive ophthalmic examination including BCVA, a computerized automatic refractor (RC-4000, Tomey, Japan), and a retinoscope (YZ24, Suzhou Liuliu Vision Technology Co., Ltd., Suzhou, China). IOLMaster 700 (i.e., biometer B; Carl Zeiss Meditec AG, Jena, Germany) was used to determine AL, flat keratometry (Kf), steep keratometry (Ks), central corneal thickness (CCT), ACD, lens thickness (LT), white-to-white (WTW) distance and pupil diameter (PD). Refraction without cycloplegia was recorded in SE. SE in diopters (D) was calculated as sphere plus half cylinder (8).

This study was approved by the Institutional Review Board (IRB) of Hohhot Chaoju Eye Hospital and performed according to the tenets of the Declaration of Helsinki. Written informed consent was obtained from participants or their parents.

### Statistical analysis

SPSS software version 20.0 (SPSS Inc., Chicago, IL, USA) was used to do the statistical analysis. Data were tested for normality with the Kolmogorov–Smirnov test. All continuous data were described as mean ± SD. A paired *t*-test was performed to investigate the change of ocular biometric indicators between the right and left eyes. Ocular biometric indicators between boys and girls were compared using an independent proportional *t*-test. One-way Analysis of Variance (ANOVA) combined with a least significant difference (LSD) *post-hoc* test was used to compare the continuous variables between four sex groups. A *p* < 0.05 was identified as statistically significant.

## Results

Table 1 shows the measures of spread for SE, ocular biometric parameters. The mean spherical equivalent refraction was −1.16 ± 2.81 D in right eyes and −1.17 + 2.81 D in left eyes (*p* = 0.861). There was no significant difference between the right and left eye in AL, CCT, ACD, LT, Ks, Kf, WTW distance, and PD (all *p* > 0.05). Herein, we present the findings of only the right eye.

**TABLE 1:** Characteristics between right and left eyes (mean ± standard deviation, SD).

	Right eyes (*n* = 64)	Left eyes (*n* = 64)	*P*
SE (D)	−1.16 ± 2.81	−1.17 + 2.81	0.861
AL (mm)	23.61 ± 1.54	23.54 ± 1.52	0.063
CCT (μm)	528.88 ± 30.26	529.23 ± 29.52	0.789
ACD (mm)	3.11 ± 0.31	3.11 ± 0.30	0.952
LT (mm)	3.42 ± 0.21	3.41 ± 0.19	0.249
Ks	43.15 ± 1.79	43.15 ± 1.75	0.988
Kf	44.64 ± 1.96	44.67 ± 2.04	0.649
WTW (mm)	11.91 ± 0.53	11.91 ± 0.56	0.876
PD (mm)	6.09 ± 0.94	6.19 ± 0.88	0.093

ACD, anterior chamber depth; LT, lens thickness; AL, axial length; CCT, central corneal thickness; Kf, keratometric readings in flattest meridian; Ks, keratometric readings in steepest meridian; PD, pupil diameter; SE, spherical equivalent; WTW, white to white diameter.

There were 28 boys and 36 girls included in the current study. Compared with boys, girls had higher height, Ks, Kf, WTW distance, and shorter AL in all twins. However, there was no significant difference in age, SE, parental SE, CCT, ACD, LT, and PD between gender groups (Table 2).

**TABLE 2:** Demographic and refractive indicators by gender (mean ± standard deviation, SD).

	Boys (*n* = 28)	Girls (*n* = 36)	*P*
Age (years)	10.46 ± 2.78	9.51 ± 2.85	0.18
**Height (cm)**	**148.96 ± 10.03**	**143.97 ± 8.6**	**0.03**
RE level of father (D)	−1.75 ± 2.75	−0.93 ± 1.79	0.15
RE level of mother (D)	−1.56 ± 2.47	−1.39 ± 2.26	0.78
SE (D)	−1.28 ± 3.33	−1.06 ± 2.37	0.75
**AL (mm)**	**24.25 ± 1.55**	**23.12 ± 1.36**	**0.03**
CCT (μm)	532.21 ± 29.32	526.35 ± 31.1	0.44
ACD (mm)	3.19 ± 0.27	3.05 ± 0.33	0.07
LT (mm)	3.37 ± 0.21	3.46 ± 0.19	0.09
**Ks**	**42.42 ± 1.66**	**43.71 ± 1.69**	**0.003**
**Kf**	**43.73 ± 1.75**	**45.33 ± 1.85**	**0.001**
**WTW** (mm)	**12.08 ± 0.62**	**11.78 ± 0.43**	**0.024**
PD (mm)	6.19 ± 0.75	6.0 ± 1.06	0.42

ACD, anterior chamber depth; LT, lens thickness; AL, axial length; CCT, central corneal thickness; Kf, keratometric readings in flattest meridian; Ks, keratometric readings in steepest meridian; PD, pupil diameter; RE, Refractive error; SE, spherical equivalent; WTW, white to white diameter. The bold values indicate statistical significance.

In the comparison of sex-paired twins, there was also no significant difference in age, and height as well as parental refraction between the four groups (all *p* > 0.05). Furthermore, SSF was found to have the lowest AL, CCT, ACD, LT, and WTW levels, while the highest Ks, and Kf levels compared with OSM, OSF, and SSM adolescents (all *p* < 0.05). There were significant differences in ACD levels [(2.99 ± 0.35) and (3.26 ± 0.15) mm, *p* = 0.033)] and Kf measures [(43.93 ± 1.64) and (42.91 ± 1.75), *p* = 0.016)] between females from SS and OS twin pairs (Table 3).

**TABLE 3:** Demographics and refractive indicators by twin sex type (mean ± standard deviation, SD).

	SSM (*n* = 20)	SSF (*n* = 28)	OSM (*n* = 8)	OSF (*n* = 8)	*P*
Age (years)	10.4 ± 2.41	9.21 ± 2.55	10.62 ± 3.74	10.62 ± 3.74	0.342
Height (cm)	148.7 ± 9.02	143.1 ± 8.12	149.63 ± 12.92	147.13 ± 10.13	0.133
RE level of father (D)	−1.97 ± 2.85	−0.86 ± 1.57	−1.21 ± 2.53	−1.21 ± 2.53	0.42
RE level of mother (D)	−1.7 ± 2.5	−1.44 ± 2.23	−1.22 ± 2.53	−1.22 ± 2.53	0.947
SE (D)	−0.96 ± 3.76	−0.99 ± 2.54	−2.09 ± 1.86	−1.31 ± 1.77	0.781
**AL** (mm)	**24.29 ± 1.62**	**22.91 ± 1.35**	**24.16 ± 1.43**	**23.89 ± 1.18**	**0.008**
CCT (μm)	530.4 ± 26.95	524.31 ± 32.08	536.75 ± 36.24	533.75 ± 27.87	0.701
**ACD** (mm)	**3.21 ± 0.27**	2.99 ± 0.35*	**3.16 ± 0.29**	3.26 ± 0.15*	**0.046**
**LT** (mm)	**3.3 ± 0.17**	**3.45 ± 0.19**	**3.55 ± 0.22**	**3.46 ± 0.21**	**0.007**
**Ks**	**42.41 ± 1.79**	**43.93 ± 1.64**	**42.45 ± 1.41**	**42.91 ± 1.75**	**0.013**
**Kf**	**43.76 ± 1.97**	**43.93 ± 1.64^†^**	**42.45 ± 1.41**	**42.91 ± 1.75^†^**	**0.001**
**WTW** (mm)	**12.1 ± 0.69**	**11.7 ± 0.41**	**12.04 ± 0.45**	**12.09 ± 0.34**	**0.034**
PD (mm)	6.28 ± 0.77	5.99 ± 1.11	6.0 ± 0.72	6.06 ± 0.95	0.766

ACD, anterior chamber depth; LT, lens thickness; AL, axial length; CCT, central corneal thickness; Kf, keratometric readings in flattest meridian; Ks, keratometric readings in steepest meridian; OSM, boys from opposite-sex twin-pairs; OSF, girls from opposite-sex twin-pairs; PD, pupil diameter; SSM, boys from same-sex twin-pairs; SSF, girls from same-sex twin-pairs; SE, spherical equivalent; WTW, white to white diameter.

* *p* = 0.033; ^†^ *p* = 0.016. The bold values indicate statistical significance.

## Discussion

There were gender differences in optical biometry measurements, while no report focuses on this topic in sex twin-pairs. In our study, girls were most likely to have lower optical biometry measurements (AL, Ks, Kf, and WTW distance). Furthermore, females from same-sex (SS) twin pairs are expected to have a lower ACD but higher keratometric readings in the flattest meridian (kf) than females from opposite-sex (OS) pairs. However, in the current study, we did not find any differences in SE, AL, CCT, LT, Ks, WTW distance, and PD between females from SS and OS twin pairs. We cannot exclude the possibility of minor differences due to the limited sample size.

It is well known that girls have shorter height and less body weight than boys including ocular development (9). While the exact mechanisms for the differences between females from SS and OS twin pairs are still unclear. Females from twin pairs had similar genetic backgrounds, and there was no statistically significant difference in the degree of RE between their parents. Variations in hormonal exposure during fetal development may have impacts on individual variability in sex-related traits (10). Girls‘ fetuses developing among opposite-sex twin pairs tend to exhibit masculinized anatomical traits as adults. On the other hand, girl fetuses developing in same-sex twin pairs tend to exhibit more feminized traits as adults. These feminized traits include various physiological and hormonal changes. This intra-uterine effect is related to the transfer of testosterone from male fetuses to adjacent fetuses (10).

Previous reports revealed that girls from SS twin pairs exhibit a decreased tooth size, as compared to girl twin pairs from OS twin pairs (11). Nevertheless, the current findings are still consistent, and negative outcomes exist for many traits, including, anthropometric measures (12) and birth weight (13).

There was a significant difference in ACD among different myopia groups (14), and ACD may be correlated with myopia. However, in the current study, we did not find any difference in SE and AL between a female in SS and OS twin pairs. A similar finding was also found in Ks parameter.

The strength of this study includes the first twin study design, as well as the comprehensive analysis of ocular biometry parameters. Notably, there were some limitations in the present study. First, we measured RE status without cycloplegia associated with pharmacological mydriasis, thus the non-significant difference between several groups should be cautious. There is a significant difference in many ocular parameters before and after cycloplegia. Herein, without cycloplegia may overestimate SE. Second, the subjects of our study were children and adolescents and mostly healthy. Herein, our findings cannot be extrapolated to middle-aged or older females in twin pairs. Moreover, we only include participants with normal or mild to moderate myopia due to limited cases with high myopia in young twin pairs. Third, this was a retrospective cross-sectional study, and some confounders such as diet, and nutrition status were insufficient. Last but not least, the sample size was relatively small, particularly in OSF and OSM groups, further large scale, prospective, and population-based study is needed to verify our findings.

## Conclusion

In sum, ocular biometry characteristics, including AL, Ks, Kf, and WTW distance differed between boys and girls in twins. Furthermore, females from SS twin pairs had lower ACD but higher Kf than those females from OS twin pairs. Collectively, our findings may help to explore the differences in ocular biometry measurements among children of different genders.

## Data availability statement

The original contributions presented in this study are included in the article/supplementary material, further inquiries can be directed to the corresponding authors.

## Ethics statement

Written informed consent was obtained from the individual(s), and minor(s)’ legal guardian/next of kin, for the publication of any potentially identifiable images or data included in this article.

## Author contributions

HL contributed to the acquisition, analysis and interpretation of the data and to the drafting and final approval of the manuscript. GZ provided technical support and conceptual advice. HL designed the study. All authors read and approved the final manuscript.
